# A mouse model of pathological small intestinal epithelial cell apoptosis and shedding induced by systemic administration of lipopolysaccharide

**DOI:** 10.1242/dmm.013284

**Published:** 2013-08-15

**Authors:** Jonathan M. Williams, Carrie A. Duckworth, Alastair J. M. Watson, Mark R. Frey, Jennifer C. Miguel, Michael D. Burkitt, Robert Sutton, Kevin R. Hughes, Lindsay J. Hall, Jorge H. Caamaño, Barry J. Campbell, D. Mark Pritchard

**Affiliations:** 1 Department of Gastroenterology, Institute of Translational Medicine, University of Liverpool, Liverpool, L69 3GE, UK; 2 Norwich Medical School, University of East Anglia, Norwich Research Park, Norwich, NR4 7TJ, UK; 3 Departments of Pediatrics, and Biochemistry and Molecular Biology, The Saban Research Institute at Children’s Hospital Los Angeles, University of Southern California, Los Angeles, CA 90027, USA; 4 NIHR Liverpool Pancreas Biomedical Research Unit, 5th Floor UCD Block, Royal Liverpool University Hospital, Daulby Street, Liverpool, L69 3GA, UK; 5 Institute of Food Research, Norwich Research Park, Colney, Norwich, NR4 7UA, UK; 6 School of Immunity and Infection, University of Birmingham, Birmingham, B15 2TT, UK

## Abstract

The gut barrier, composed of a single layer of intestinal epithelial cells (IECs) held together by tight junctions, prevents the entrance of harmful microorganisms, antigens and toxins from the gut lumen into the blood. Small intestinal homeostasis is normally maintained by the rate of shedding of senescent enterocytes from the villus tip exactly matching the rate of generation of new cells in the crypt. However, in various localized and systemic inflammatory conditions, intestinal homeostasis can be disturbed as a result of increased IEC shedding. Such pathological IEC shedding can cause transient gaps to develop in the epithelial barrier and result in increased intestinal permeability. Although pathological IEC shedding has been implicated in the pathogenesis of conditions such as inflammatory bowel disease, our understanding of the underlying mechanisms remains limited. We have therefore developed a murine model to study this phenomenon, because IEC shedding in this species is morphologically analogous to humans. IEC shedding was induced by systemic lipopolysaccharide (LPS) administration in wild-type C57BL/6 mice, and in mice deficient in TNF-receptor 1 (*Tnfr1^−/−^*), *Tnfr2* (*Tnfr2^−/−^*), nuclear factor kappa B1 (*Nfκb1^−/−^*) or *Nfĸb2* (*Nfĸb2^−/−^*). Apoptosis and cell shedding was quantified using immunohistochemistry for active caspase-3, and gut-to-circulation permeability was assessed by measuring plasma fluorescence following fluorescein-isothiocyanate–dextran gavage. LPS, at doses ≥0.125 mg/kg body weight, induced rapid villus IEC apoptosis, with peak cell shedding occurring at 1.5 hours after treatment. This coincided with significant villus shortening, fluid exudation into the gut lumen and diarrhea. A significant increase in gut-to-circulation permeability was observed at 5 hours. TNFR1 was essential for LPS-induced IEC apoptosis and shedding, and the fate of the IECs was also dependent on NFκB, with signaling via NFκB1 favoring cell survival and via NFκB2 favoring apoptosis. This model will enable investigation of the importance and regulation of pathological IEC apoptosis and cell shedding in various diseases.

## INTRODUCTION

The gut barrier consists of a single layer of intestinal epithelial cells (IECs) and the tight junctions between them. It allows absorption of nutrients from the intestinal lumen into the circulation, while preventing the entry of injurious microorganisms, toxins and antigens. In the small intestine, IECs are generated in the crypt, migrate up the villus and are shed at the villus tip (Leblond and Stevens, 1948). In mice, which exhibit whole IEC shedding similar to that which occurs in humans (Bullen et al., 2006), ∼1400 IECs are estimated to be shed in this way from a single villus tip per day (Potten and Loeffler, 1990). The small intestine therefore has one of the highest cell turnover rates in the body, with an estimated 10^11^ and 2×10^8^ cells being shed per day from the small intestine of humans and mice, respectively (Potten and Loeffler, 1990). During the process of physiological cell shedding, the highly coordinated process of tight junction rearrangement that is required to allow the detachment and release of IECs from the epithelium maintains the gut barrier (Madara, 1990).

In various inflammatory conditions, however, the loss of IECs from the villus exceeds the rate of epithelial generation in the crypt. This process, which we have termed ‘pathological IEC shedding’ remains poorly understood. Such pathological IEC shedding might represent the earliest intestinal injury in a variety of intestinal diseases and is likely to have important consequences, potentially resulting in gap formation in the epithelium, permeability defects and villus shortening (villus atrophy). Indeed, increased numbers of shedding IECs with corresponding focal permeability defects and epithelial gaps have been observed in inflammatory bowel disease (IBD), including both Crohn’s disease (CD) and ulcerative colitis (UC) (Kiesslich et al., 2012; Liu et al., 2011). It has also been shown that individuals at high risk of developing IBD exhibit increased gastrointestinal permeability (Hollander et al., 1986). Similarly, IL-10-deficient mice exhibit increased small intestinal permeability prior to the development of spontaneous colitis. In this animal model, colitis severity can be markedly reduced by administering a specific pharmacological inhibitor that reduces small intestinal permeability by preventing the opening of tight junctions, and is prevented completely by rearing animals in germ-free conditions (Arrieta et al., 2009). This suggests a crucial link between small intestinal permeability, luminal antigens and the development of chronic colitis.

**TRANSLATIONAL IMPACT**

**Clinical issue**

Epithelial cell loss and defects in the epithelial barrier are common early events in the pathogenesis of acute intestinal or diarrheal diseases as well as chronic intestinal inflammatory disorders, such as Crohn’s disease and ulcerative colitis. Increased intestinal epithelial cell (IEC) shedding, which is potentially associated with increased gut permeability, is thought to be a key contributor to these initial injuries. However, the molecular events underlying IEC shedding and disruption of intestinal barrier integrity during inflammatory disease states remain poorly understood. A robust animal model would greatly facilitate in-depth investigation of these processes. Mice exhibit a morphologically analogous form of small intestinal villus epithelial cell shedding to that observed in humans and have the potential to allow important mechanistic insights because of the availability of established transgenic and conditional mutant models.

**Results**

Utilizing the inflammatory response induced by systemic administration of lipopolysaccharide (LPS) in mice, the authors developed a new model for the investigation of IEC shedding. A threshold dose of ≥0.125 mg LPS/kg body weight delivered by intraperitoneal injection induced rapid and dynamic villus IEC apoptosis and shedding, which peaked at 1.5 hours post-administration. This coincided with significant villus shortening, fluid exudation into the gut and the onset of diarrhea. Activation of caspase-3 occurred concomitantly with IEC shedding in virtually all affected cells, suggesting that apoptosis is triggered within the epithelium prior to cells being shed. By examining the responses to LPS in transgenic mice, the authors showed that TNFR1 is essential for the induction of apoptosis and shedding. Furthermore, the fate of IECs is dependent on NFκB signaling, with signaling by NFκB1 favoring cell survival and signaling via NFκB2 favoring apoptosis.

**Implications and future directions**

Systemic LPS administration in mice provides an effective and reliable trigger of pathological IEC shedding that parallels that observed in human intestinal diseases. The authors’ detailed characterization of affected mice confirms that villus epithelial apoptosis and cell shedding occurs with acute fluid exudation into the gut, leading to diarrhea. Furthermore, they provide evidence that TNFR1 and NFκB2-dominant signaling pathways are important in IEC apoptosis and shedding. By conducting an in-depth investigation of the kinetics, dose response and signaling mechanisms underlying these processes, they have established a novel and robust model that will facilitate further investigation of epithelial gap formation and barrier dysfunction in the inflamed intestinal epithelium. Further studies using this model are likely to provide insights into disease pathogenesis and support the development of more targeted treatments for inflammatory disorders.

Although recombinant tumor necrosis factor (TNF) has been previously shown to induce pathological intestinal villus epithelial cell shedding in mice (Kiesslich et al., 2007), the exogenous administration of this cytokine in isolation does not reflect the complexity of the mammalian inflammatory response that is present in most disease states. In addition, the concentrations of exogenous TNF required to induce IEC shedding are higher than found *in vivo*, making such experiments expensive and possibly yielding artefactual results. We have also found TNF to be an inconsistent stimulus of IEC shedding. We therefore sought to find a simple, inexpensive, rapid, reproducible and pathologically relevant stimulus to investigate the process of IEC shedding in detail in an animal model.

Lipopolysaccharide (LPS) is an integral component of Gram-negative bacteria and is a potent activator of the innate immune system. It represents a pathogen-associated molecular pattern (PAMP) recognized by Toll-like receptor 4 (TLR4) (Beutler et al., 2001), which initiates a systemic inflammatory response, with nuclear factor kappa B (NFκB) signaling pathways playing a central role in cell responses downstream of both TLR (Chow et al., 1999) and subsequent cytokine receptor ligation (Jacque et al., 2005). We therefore hypothesized that the mammalian systemic inflammatory response was capable of causing IEC apoptosis and shedding at the villus tip when triggered by LPS, and that this occurred prior to the onset of apoptosis in the crypt.

We have therefore examined in detail the earliest phase of LPS-induced murine gut injury. We demonstrate that intraperitoneally administered LPS is a simple, rapid and consistent stimulus of villus IEC apoptosis and shedding in the murine small intestine and that this occurs several hours prior to the onset of crypt apoptosis. This early response coincides with fluid effusion into the small intestinal lumen and diarrhea. We have subsequently characterized the dose response and the kinetics of this highly dynamic phenomenon. Using knockout mouse models, we have found that TNFR1-mediated signaling is essential for these events, with an NFκB2-dominant response favoring apoptosis. These data provide interesting insights into the control of IEC homeostasis in inflammatory disease, because the NFκB2 pathway has not previously been linked to IEC apoptosis and shedding.

## RESULTS

### Systemic LPS caused clinical signs and gross pathological changes from 1.5 hours, with fluid exudation into the intestinal lumen

**Fig. 1.**
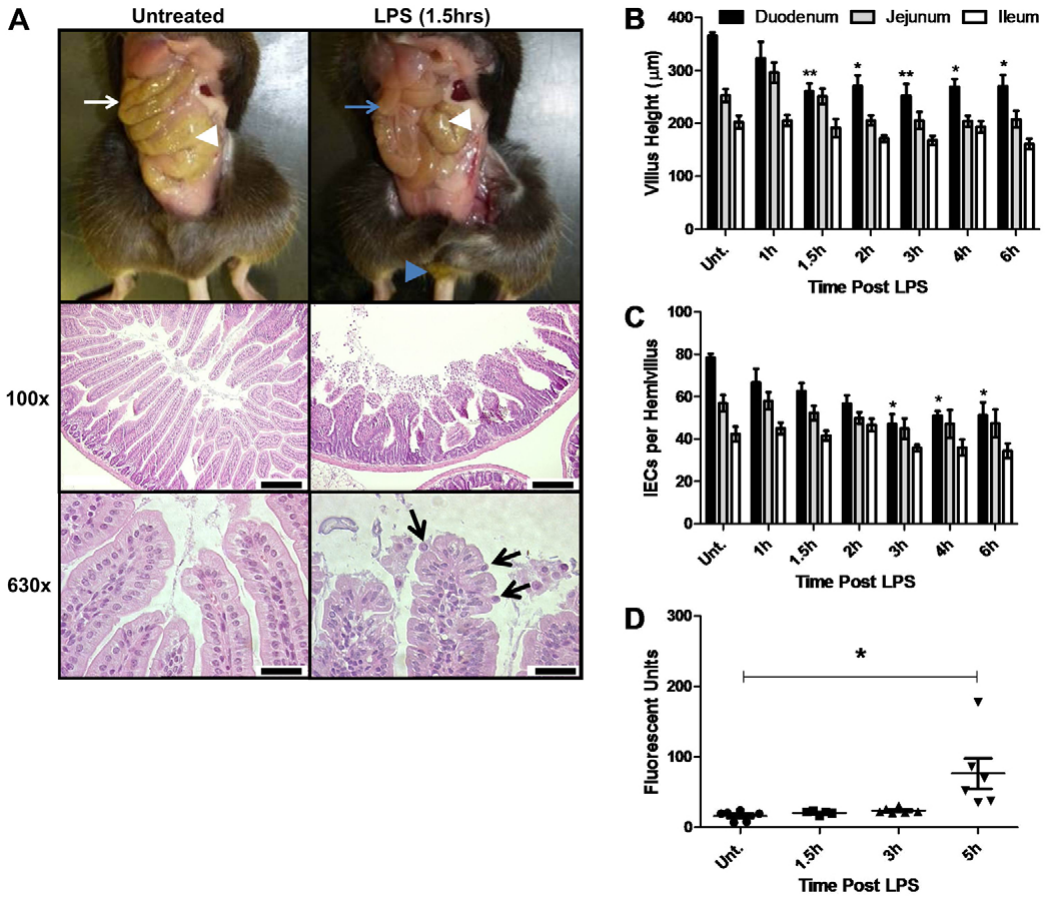
**LPS at 10 mg/kg body weight caused acute diarrhea, villus epithelial cell loss, villus shortening and increased gut permeability**. (A) Small intestine of an untreated control (white arrow) and a WT mouse 1.5 hours after LPS treatment exhibiting a fluid-filled small intestine devoid of digesta (blue arrow) with diarrheic feces at anus (blue arrowhead). Caeca are indicated by white arrowheads. H&E-stained sections of duodenum (100×) show normal villi in an untreated control and shortened, blunted and clubbed villi with shed IECs in the lumen at 1.5 hours post-LPS. Scale bars: 200 μm. Villus tips (630×) are shown in an untreated control, and shedding IECs are seen at 1.5 hours post-LPS (arrows). Scale bars: 25 μm. (B) Villus heights for duodenum, jejunum and ileum (*n*=6). (C) Hemivillus IEC counts from base to apex for duodenum, jejunum and ileum (*n*=6). (D) Plasma fluorescence measured after 10 mg PELPS/kg body weight and gavage of FD4. *n*=6, one outlier excluded at 1.5 hours. *P<0.05, **P<0.01. Comparisons by ANOVA in A, and Kruskal-Wallis in B-D.

To establish the time-dependent intestinal effects of LPS, we administered 10 mg phenol-extracted LPS (PE-LPS)/kg body weight by intraperitoneal (i.p.) injection to adult female wild-type (WT) mice, and euthanized them after 1, 1.5, 2, 3, 4 and 6 hours. Diarrhea was observed from 1.5–2 hours. At necropsy, there was serosal pallor of the small intestine (Fig. 1A), which exhibited distension with watery yellow fluid. These observations showed that LPS caused acute fluid exudation into the gut lumen with acute onset diarrhea.

### LPS caused small intestinal villus IEC loss and shedding from 1.5 hours

We performed histopathological examination of hematoxylin and eosin (H&E)-stained sections to characterize intestinal injury. At 1.5 hours (Fig. 1A) there was marked villus shortening, clubbing and blunting. IECs at the villus tip exhibited variable separation and detachment from neighboring cells, often with a teardrop morphology and an apically positioned nucleus (consistent with cell shedding and apoptosis). Large numbers of shed IECs were present within the lumen. Comparable injury was not observed in the stomach, colon or other organs investigated (supplementary material Fig. S1). These observations suggest that LPS causes rapid and specific small intestinal villus epithelial injury, and that peak shedding correlates with the onset of clinical diarrhea.

### LPS caused rapid villus shortening with IEC loss in the duodenum, jejunum and ileum

Because villus shortening is commonly utilized as a measure of small intestinal damage, we measured villus heights after LPS administration. In the duodenum at 1.5 hours after LPS administration, mean villus height was reduced by 29% (Fig. 1B) to 260.5±15.0 μm compared with villi from untreated mice (365.9±6.6 μm) (*P*<0.01: ANOVA). The reduction in villus height was still evident in treated versus non-treated mice through to 6 hours post-LPS (all *P*<0.05: ANOVA). A similar trend was also observed in both the jejunum and ileum, but differences did not reach statistical significance.

Villus shortening was associated with lower numbers of IECs lining the duodenal villi, with a 21% reduction in mean cell number observed at 1.5 hours post LPS administration compared with controls (62.7±3.7 versus 78.5±1.8 IECs in untreated mice), reaching significance at 3 hours, at which point a 40% decrease was observed (47.0±4.7 IECs, *P*<0.05: Kruskal-Wallis) (Fig. 1C). This correlated with large numbers of shed IECs within the intestinal lumen, suggesting that cell shedding occurs contemporaneously with villus shortening.

### LPS significantly increased gut-to-circulation permeability by 5 hours

In order to measure gut-to-circulation permeability, mice were administered fluorescein-isothiocyanate-conjugated dextran (FD4) by oral gavage, followed by 10 mg LPS/kg body weight. At 5 hours post-LPS (5 hours FD4), there was a fivefold increase (*P*<0.05: Kruskal-Wallis) in plasma fluorescence, at 76.3±21.7 fluorescent units (Fig. 1D), compared with untreated mice (16.4±2.9 at 5 hours FD4), suggesting that gut barrier dysfunction allows large molecules to enter the bloodstream at around 5 hours post-LPS.

### LPS caused activation of caspase-3 with concomitant apoptosis and shedding of villus IECs, and relatively spared the crypts

**Fig. 2.**
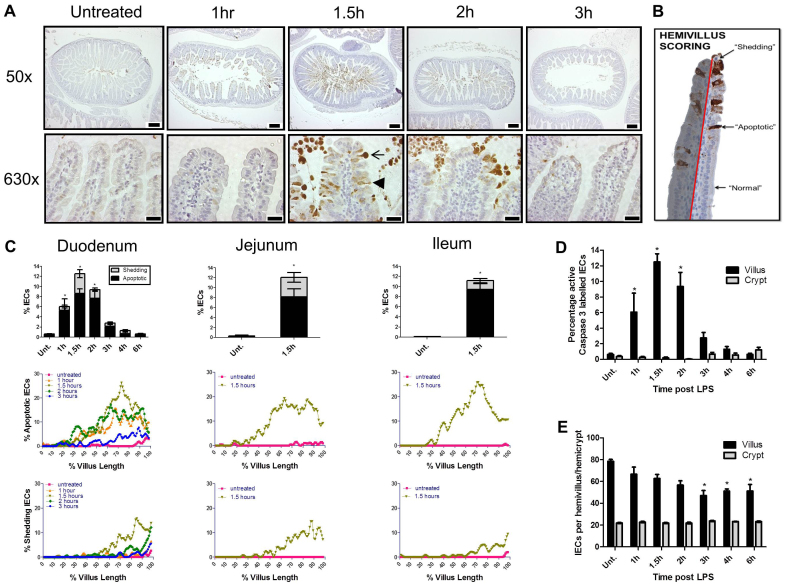
**LPS caused activation of caspase-3 with concomitant apoptosis and shedding of villus IECs, and relatively spared the crypts**. (A) Duodenal sections labeled for active caspase-3 by IHC. Arrowhead indicates a positively labeled apoptotic IEC with unaltered morphology, and arrow indicates a positively labeled IEC with shedding morphology. Scale bars: 200 μm (50×), 25 μm (630×). (B) Example of a duodenal villus 1.5 hours following administration of 10 mg PELPS/kg body weight to a C57BL/6 female mouse. Individual cells were counted along the epithelial monolayer lining one side of a villus (delineated by red line) from base to tip, and therefore referred to as a ‘hemivillus’. Cells were categorized as ‘normal’, ‘apoptotic’ or ‘shedding’. A total of 18–20 hemivilli were analyzed for each intestinal segment for each individual animal. (C) Quantification of apoptotic and shedding IECs in the duodenum, jejunum and ileum (bar graphs), and cell positional quantification of ‘apoptotic’ or ‘shedding’ IECs in the duodenum, jejunum and ileum along villus length (line graphs; 0% villus length represents the villus base, 100% represents the villus tip). (D) Quantification of active-caspase-3-positive cells in villus versus crypt IECs. (E) Villus versus crypt IEC counts. *n*=6 female mice/group; *P<0.05, comparisons by ANOVA.

To investigate the type of cell death responsible for IEC shedding and loss from the villus, we performed immunohistochemistry (IHC) for active caspase-3. Large numbers of villus IECs exhibited positive immunolabeling as early as 1 hour, with almost universal immunolabeling of shed cells seen within the small intestinal lumen (Fig. 2A). Villus IECs were quantified by microscopy as ‘apoptotic’ or ‘shedding’ (as defined in Materials and Methods and summarized in Fig. 2B) and expressed as a percentage of total villus IECs counted.

Maximal active caspase-3 labeling of 12.5±1.7% villus IECs was found in the duodenum 1.5 hours after LPS (Fig. 2C), representing a 21-fold increase compared with untreated mice (0.6±0.2%, *P*<0.05: Kruskal-Wallis). Comparable IEC apoptosis and cell shedding were also observed at 1.5 hours after LPS treatment in the jejunum and ileum (12.1±2.4% and 11.2±1.3%, respectively). We therefore concluded that LPS caused dynamic villus IEC apoptosis and shedding, and that this occurred relatively uniformly throughout the small intestine. The almost universal positive labeling of IECs undergoing shedding additionally suggests that activation of the terminal pathway of apoptosis occurs prior to shedding in this model, rather than being triggered by detachment as occurs during the process of anoikis. Interestingly, crypt IEC apoptosis as interpreted by active caspase-3 IHC did not show a comparable magnitude of increase to that observed in villi at 1.5 hours (Fig. 2D), although there was an ∼threefold increase by 6 hours post-LPS (1.2±0.3% versus 0.4±0.1% in untreated). Accordingly, crypt counts did not alter significantly throughout the time course studied (Fig. 2E), in contrast to villus IEC counts.

### LPS-induced apoptosis and cell shedding increased towards the villus tip

Administration of LPS (10 mg/kg body weight) increased the number of apoptotic and/or shedding IECs with similar distribution along the villus axis in the duodenum, jejunum and ileum (Fig. 2C). Apoptosis was markedly increased in the apical 50% of the villus, particularly at 1.5 hours, with a sharp increase in IEC shedding being observed at the villus tip, compared with controls.

### LPS caused maximal apoptosis and shedding at a threshold dose

**Fig. 3.**
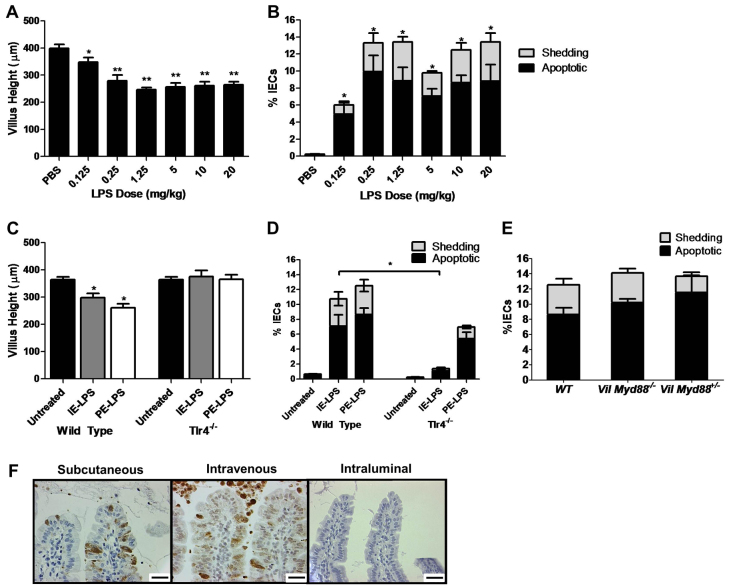
**LPS caused a plateau in apoptosis and shedding at a threshold dose through TLR4 signaling peripheral to epithelial cells**. (A) Heights of duodenal villi 1.5 hours after PE-LPS administration at the indicated doses and (B) quantification of percentage apoptotic and shedding IECs by assessment of duodenal sections labeled for active caspase-3 by IHC. *n*=6 female mice/group for PBS, 0.125 mg/kg and 10 mg/kg, and *n*=4 female mice for all other groups. (C) Villus heights in WT or *Tlr4^−/−^* mice 1.5 hours after PE-LPS or IE-LPS treatment at 10 mg/kg, and (D) quantification of IEC apoptosis and shedding in IHC-labeled duodenal sections (*n*=6). (E) Quantification of apoptotic and shedding IECs in WT (*n*=6), *Vil-Cre Myd88^−/−^* (*n*=3) and *Vil-Cre Myd88^+/−^* (*n*=3) mice by assessment of duodenal sections labeled for active caspase-3 by IHC. (F) Small intestinal villi from WT female mice 1.5 hours post-PE-LPS by subcutaneous (s.c.) or intravenous (i.v.) routes exhibited villus IEC apoptosis and shedding, but not when LPS was instilled intraluminally [active caspase-3 IHC, duodenum shown for s.c. and i.v. LPS (*n*=3 per group), ileum shown for intraluminal administration instilled with 1 mg/ml LPS (*n*=4 per group)]. Scale bars: 25 μm. *P<0.05, **P<0.01. Comparisons by ANOVA in A-C, and by Kruskal-Wallis and pairwise comparison of LPS-treated mice only in D.

We administered 0.125–20 mg PE-LPS/kg body weight to WT mice and euthanized them after 1.5 hours to test whether LPS-induced IEC apoptosis and shedding was dose dependent. LPS at 0.125 mg/kg caused a minimal (5%) reduction in villus height [348.1±17.1 μm versus 399.0±35.5 μm in vehicle-treated control mice (Fig. 3A)] but with a tenfold observed increase in IEC apoptosis and cell shedding at 6.0±1.7% (*P*<0.05: ANOVA) (Fig. 3B). LPS doses ≥0.25 mg/kg body weight caused ∼30% reduction in villus height compared with controls, and IEC apoptosis and cell shedding of ∼12%. We concluded that LPS-induced small intestinal injury is initiated by a threshold dose of ∼0.125 mg/kg body weight. Villus IECs therefore seem to be extremely sensitive to LPS-induced apoptosis and cell shedding, whereas concomitant villus shortening only occurs at higher dosages of LPS.

### LPS purity did not significantly affect IEC apoptosis and shedding

To assess whether the LPS purification and/or extraction method altered IEC apoptosis and shedding, we administered high-purity ion-exchange chromatography extracted LPS (IE-LPS; 10 mg/kg body weight) to WT mice for 1.5 hours. This preparation caused similar villus shortening to 10 mg PE-LPS/kg body weight (298.0±39.1 μm compared with 260.5±36.8 μm, respectively) (Fig. 3C). IEC apoptosis and shedding post IE-LPS administration were also significantly increased (10.8±2.8%) compared with untreated WT, as observed for PE-LPS (12.5±1.7%) (Fig. 3D).

### LPS-induced apoptosis and cell shedding was significantly decreased in *Tlr4^−/−^* mice and was due to TLR ligation peripheral to IECs

Because TLR4 is necessary for the innate immune system to respond to LPS (Beutler et al., 2001), we investigated whether *Tlr4^−/−^* mice would exhibit LPS-induced small intestinal injury, to exclude the possibility of alternative mechanisms. IE-LPS (10 mg/kg body weight) caused negligible change in villus height in *Tlr4^−/−^* mice (Fig. 3C) and negligible IEC apoptosis and shedding compared with WT mice (Fig. 3D). However, it should be noted that, when *Tlr4^−/−^* mice were administered 10 mg PE-LPS/kg body weight, although this resulted in negligible change in villus height compared with untreated *Tlr4*^–/–^ mice (Fig. 3C), moderate IEC apoptosis and shedding of 7.0±1.0% IECs was seen (Fig. 3D). To exclude the possibility that IEC apoptosis and shedding was affected by direct TLR ligation in IECs, we additionally tested the response of *Villin-Cre* (*Vil-Cre*) *Myd88^−/−^* mice, which lack the TLR signaling adapter molecule Myd88 in IECs, to systemic administration of 10 mg PE-LPS/kg body weight. These mice showed very comparable amounts of apoptosis and shedding (14.1±1.1% IECs) to their WT and heterozygous counterparts (Fig. 3E). Furthermore, we tested the small intestinal response to LPS by alternative routes of administration at 1.5 hours. We found that, although intraperitoneal, intravenous or subcutaneous LPS administration caused IEC apoptosis and shedding, when LPS was delivered directly into the lumen of a ligated segment of small intestine in terminally anesthetized WT mice, this did not initiate apoptosis and shedding (Fig. 3F).

These results suggest that LPS-induced small intestinal injury is dependent on TLR4 signaling peripheral to IECs and that additional bacterial components in PE-LPS cause IEC shedding via TLR4-independent mechanisms.

### *Nfĸb1^−/−^* mice were more sensitive, and *Nfĸb2^−/−^* mice more resistant, to LPS-induced intestinal injury

**Fig. 4.**
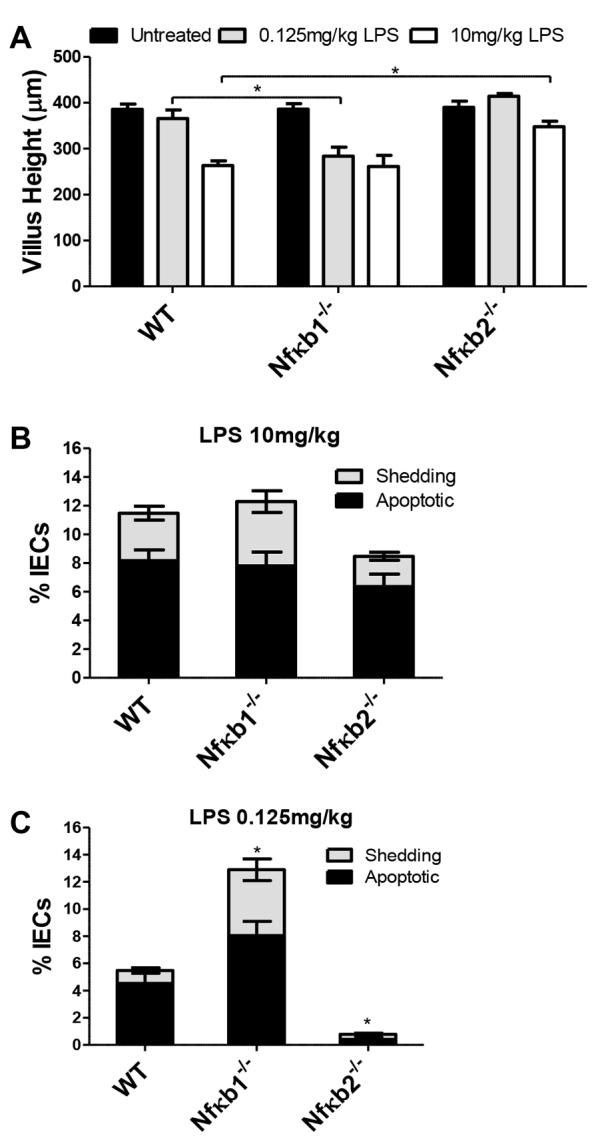
***Nfĸb1^−/−^* mice were more sensitive, and *Nfĸb2^−/−^* mice more resistant, to LPS-induced small intestinal injury.** (A) Villus heights of duodenal villi 1.5 hours after 0.125 mg/kg or 10 mg/kg PE-LPS in WT, *Nfκb1^−/−^* and *Nfκb2^−/−^* mice (comparisons between genotypes within same dosage groups only). (B,C) Quantification of apoptotic and shedding IECs in duodenal sections labeled for active caspase-3 in WT, *Nfĸb1^−/−^* and *Nfĸb2^−/−^* mice 1.5 hours after 10 mg/kg PE-LPS (B) and after 0.125 mg/kg PE-LPS (C). *n*=12–14 (male and female equally represented); *P<0.05, comparisons by Kruskal-Wallis.

NFκB is a major transcriptional regulator downstream of TLR4. We therefore administered PE-LPS to *Nfĸb1^−/−^* and *Nfĸb2^−/−^* mice, to establish whether either of these subunits, integral to the canonical and non-canonical NFĸB signaling pathways, respectively, is necessary for LPS-induced small intestinal injury. After administration of 10 mg LPS/kg body weight, similar villus shortening was seen in *Nfĸb1^−/−^* and WT mice (Fig. 4A). This genotype also showed similar IEC apoptosis and shedding to WT mice (Fig. 4B). In contrast, *Nfĸb2^−/−^* mice showed a significantly attenuated 11% villus height reduction in treated versus untreated, compared with 32% in treated versus untreated WT (*P*<0.05: Kruskal-Wallis) (Fig. 4A), and reduced IEC apoptosis and shedding compared with WT mice (Fig. 4B). Interestingly, when 0.125 mg LPS/kg body weight was administered, *Nfĸb1^−/−^* mice showed greater villus shortening (supplementary material Fig. S2) of 27% in treated versus untreated (*P*<0.05: Kruskal-Wallis) compared with 5% shortening in treated versus untreated WT mice (Fig. 4A), and significantly greater IEC apoptosis and shedding at 12.9±1.7% IECs (*P*<0.05: Kruskal-Wallis) (Fig. 4C) compared with WT mice (5.5±1.3%). IEC apoptosis and shedding in *Nfĸb2^−/−^* mice administered 0.125 mg LPS/kg body weight were negligible (0.8±0.2% IECs; *P*<0.05: Kruskal-Wallis) compared with WT mice. Together, these results suggest that LPS-induced intestinal injury is dependent on NFκB2, whereas NFκB1 might be necessary to suppress IEC apoptosis and shedding.

### LPS induced a significant increase in small intestinal *Tnf* mRNA

**Fig. 5.**
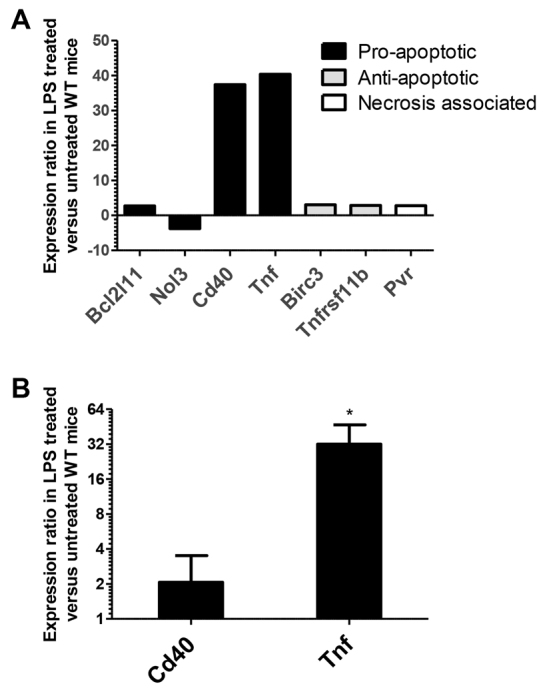
**LPS induced a significant increase in small intestinal *Tnf* mRNA.** (A) qPCR array data for selected genes that exhibited a ≥twofold expression ratio out of 89 cell-death-pathway-associated genes assessed in pooled epithelial enriched extracts. (B) Mean gene expression ratio of PE-LPS-treated (10 mg/kg, 1.5 hours) versus untreated WT female mice for *Cd40* and *Tnf. n*=4, *P<0.05: randomization test.

Because activation of caspase-3 does not categorically confirm that cell death has occurred by apoptosis, we performed an array analysis of 89 genes associated with various cell death pathways in PE-LPS-treated compared with untreated WT animals. We found that LPS predominantly altered expression of genes associated with apoptosis, rather than those associated with autophagy or necrosis (Fig. 5A). *Tnf* and *Cd40* showed marked upregulation, and we therefore analyzed these two proapoptotic genes by quantitative PCR (qPCR) using triplicate samples from individual animals (Fig. 5B). This showed a mean normalized gene expression ratio of +32.0 for *Tnf* mRNA (*P*<0.05: randomization test). qPCR also showed a non-significant increase of +2.1 for *Cd40*. These data, in conjunction with histopathological findings and activation of caspase-3, suggest that apoptosis is the predominant form of cell death occurring in LPS-induced small intestinal injury.

### TNF caused small intestinal injury equivalent to LPS

**Fig. 6.**
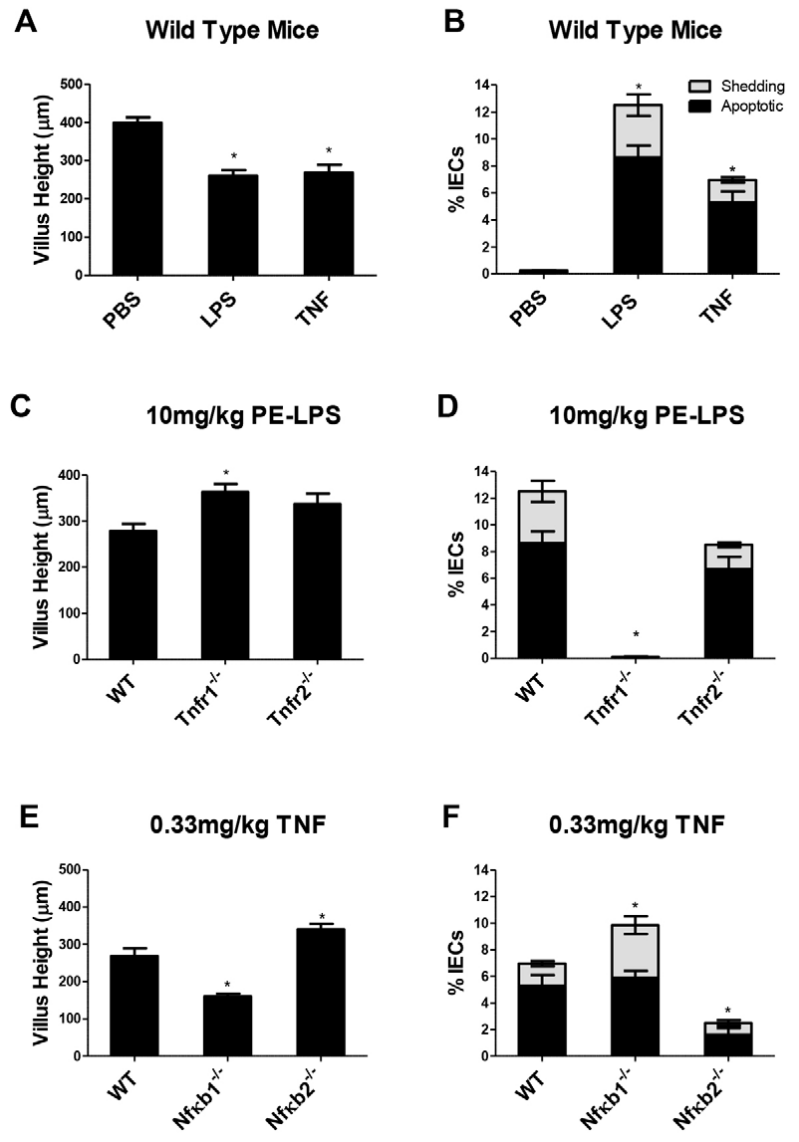
**LPS induced small intestinal IEC apoptosis and shedding via TNF and TNFR1, and was regulated by NFκB.** Villus heights and IEC apoptosis and shedding 1.5 hours after 10 mg/kg PE-LPS or 0.33 mg/kg TNF in WT mice (A,B), after 10 mg/kg PE-LPS in WT, *Tnfr1^−/−^* and *Tnfr2^−/−^* mice (C,D), and after TNF administration in WT, *Nfκb1^−/−^* and *Nfĸb2^−/−^* mice (E,F). *P<0.05; *n*=4–6 mice per group; comparisons by ANOVA, except in D (Kruskal-Wallis).

Because TNF is a key mediator of endotoxic shock, and was markedly upregulated at the mRNA level in our array, we tested whether TNF would cause comparable enteric injury to LPS. At 1.5 hours, TNF (0.33 mg/kg body weight; i.p.) caused equivalent duodenal villus shortening (Fig. 6A) to that seen with 10 mg PELPS/kg body weight (268.4±20.9 μm and 260.5±15.0 μm, respectively). Although less IEC apoptosis and shedding were observed with TNF (7.0±1.0% IECs) compared with 10 mg PELPS/kg body weight (12.5±1.7% IECs) (Fig. 6B), this reflects a faster small intestinal response to exogenously administered TNF (supplementary material Fig. S3) than caused by LPS.

These data, together with significant induction of intestinal *Tnf* mRNA, suggest that TNF is central in the pathogenesis of LPS-induced small intestinal injury.

### *Tnfr1^−/−^* mice were completely resistant to LPS-induced apoptosis and cell shedding

We decided to further examine the role of TNF by testing whether the TNF receptors TNFR1 (p55) or TNFR2 (p75) were required to cause LPS-induced gut injury. When *Tnfr1^−/−^* mice were administered 10 mg PE-LPS/kg body weight for 1.5 hours, there was no villus shortening (Fig. 6C) and significantly less IEC apoptosis and shedding were seen relative to WT animals (0.1±0.1%, *P*<0.05: Kruskal-Wallis; Fig. 6D). By contrast, the same dose administered to *Tnfr2^−/−^* mice caused 68% of the response caused in WT, at 8.5±1.0% IEC apoptosis and shedding, although this did not cause a significant change in villus height (Fig. 6C). These findings suggest that TNFR1 signaling is required to drive LPS-induced IEC apoptosis and shedding, with potential enhancement by TNFR2.

### *Nfĸb1^−/−^* mice were highly sensitive, and *Nfĸb2^−/−^* resistant, to TNF-induced small intestinal injury

NFκB is also a major exponent of downstream TNFR signaling. We therefore administered TNF to *Nfĸb1^−/−^* and *Nfĸb2^−/−^* mice. *Nfĸb1^−/−^* mice were highly sensitive to TNF (supplementary material Fig. S2) and exhibited a significant reduction in villus height compared with TNF-treated WT mice (villus heights of 160.1±7.3 and 268.4±20.9 μm, respectively; *P*<0.05: ANOVA; Fig. 6E). This correlated with increased IEC apoptosis and shedding in *Nfĸb1^−/−^* versus WT mice (9.9±0.7 and 7.0±1.0% IECs, respectively; *P*<0.05: ANOVA). Conversely, *Nfĸb2^−/−^* mice were resistant to the TNF-induced reduction in villus height (340.1±15.1 μm) and IEC apoptosis and shedding (2.5±0.7%) compared with similarly treated WT animals (both *P*<0.05; Fig. 6F).

These data suggest that IEC apoptosis and shedding in response to LPS or TNF are regulated by a common NFκB signaling pathway, being suppressed by NFκB1 but promoted by NFκB2.

## DISCUSSION

We present a detailed study of acute LPS-induced murine gut injury. Systemic LPS administration caused rapid IEC apoptosis and shedding in the murine small intestinal villus, and this resulted in shortening of the villus, fluid effusion into the small intestinal lumen and diarrhea.

We have characterized the dose response and kinetics of this highly dynamic phenomenon and demonstrate that it occurs within a tightly defined time period. All regions of the small intestine responded in a similar manner to LPS and in all cases apoptosis and cell shedding occurred in the apical 50% of the villus rather than exclusively at the tip. Using knockout mouse models, we confirmed that TLR4 signaling peripheral to the IEC was required, and that TNFR1-mediated signaling was essential for these events, with an NFκB2-dominant response favoring apoptosis.

Although there is an abundance of literature describing small intestinal crypt apoptosis several hours after the induction of endotoxic or septic shock (Cinel et al., 2002; Coopersmith et al., 2003; Guma et al., 2011), we present novel observations that the villus IECs respond much more rapidly than crypt IECs, and exhibit exquisite susceptibility to apoptosis and cell shedding in the earliest phases following LPS administration. The only other study to date that has examined small intestinal villus epithelial shedding in response to LPS studied this response from 5.5 hours post-LPS-administration by *in vivo* confocal microscopy, correlating gap formation with gut barrier dysfunction (Lai et al., 2013). This highlights the necessity of a detailed study of the kinetics of this response because, in our model, we found that the number of shedding events was profoundly reduced by 4 hours after LPS administration and the maximum response was observed as early as 1.5 hours. We found that, although multiple organ failure in the context of endotoxic shock has been extensively investigated, most commonly by biochemical parameters, obvious organ injury in terms of apoptosis was confined to the small intestine at the early time points examined herein. The reasons underlying this selective early injury to the villus IECs of the murine small intestine are not entirely clear. However, this phenomenon has been attributed to the greater sensitivity of the intestinal epithelium to mitochondrial damage than epithelia found in other commonly injured organ systems such as the lung. Interestingly, in the feline septic shock model in which this was demonstrated, other obvious hemodynamic derangements to which this effect might have been attributed, such as hypotension, intestinal hypoperfusion and hypoxia, were shown not to be responsible (Julian et al., 2011). Additionally, in our own studies, this small intestinal injury occurred by 1.5 hours not only when LPS was administered intraperitoneally, but also when given intravenously or subcutaneously, suggesting that this injury is not due to a localized phenomenon.

Clinically, we found that the onset of diarrhea correlated temporally with IEC apoptosis and shedding. This suggests that the shedding of IECs permits the net movement of fluid from the plasma into the intestinal lumen. This might be directly due to the rapid and uncoordinated shedding of IECs, potentially in conjunction with increased vascular permeability, which causes disruption of both tight junctions and the paracellular space. Our own studies have previously shown that barrier loss in the intestine occurs at sites of excessive cell shedding (Kiesslich et al., 2012), and that the direction of fluid movement through epithelial defects is highly dependent on the osmotic and hydrostatic gradients across the epithelium. The concept of acute fluid exudation into the intestinal lumen after the administration of inflammatory stimuli has also been recognized in other studies utilizing LPS or TNF (Gadjeva et al., 2007; Kiesslich et al., 2012). It was not until 5 hours after LPS administration, however, that we found movement of larger molecules (FD4) from the lumen to the plasma. This is in agreement with findings from *in vivo* confocal microscopy that, from 5.5 hours after LPS administration, FD4 entered cell-free gaps and paracellular spaces (Lai et al., 2013).

In our model, a high-purity preparation of LPS caused villus IEC apoptosis and shedding through a TLR4-dependent mechanism, but PE-LPS of lower purity was capable of inducing a moderate response via TLR4-independent mechanisms, most likely due to ligation of alternative TLRs by residual impurities such as bacterial RNA. In support of other PAMPs causing this type of response, another recent study has demonstrated that the apoptosis in the intestinal villus by the viral PAMP, double-stranded RNA, occurred via TLR3 (McAllister et al., 2013). TLR3, in contrast to other TLRs, signals exclusively via the TRIF pathway rather than the Myd88 pathway (Kawai and Akira, 2011). As such, this agonist represents an unusual type of inflammatory response. It caused apoptosis by a TRIF-dependent and TNF-independent mechanism, which peaked at 2 hours post-administration, possibly reflecting delayed activation of the TRIF pathway compared with the Myd88 pathway (Pålsson-McDermott and O’Neill, 2004).

Most previous studies have found only low-level expression of TLR4 in IECs (Abreu, 2010). Therefore, rather than occurring in IECs themselves, initial recognition of systemically delivered LPS likely occurs via TLR4 ligation in monocytes and macrophages, which in turn rapidly secrete cytokines, including TNF (Beutler et al., 1985). To confirm this mechanism in our model, we administered LPS by i.p. injection to *Vil-Cre Myd88^−/−^* mice that specifically lacked intestinal TLR signal transduction (Fig. 3E). They showed comparable IEC apoptosis and shedding to their heterozygous counterparts and WT mice of the same strain, demonstrating that peripheral TLR signaling is required for LPS-induced small intestinal injury.

LPS signaling is additionally dependent on delivery of LPS to the cell membrane in a bioactive form by lipopolysaccharide binding protein (LBP) and the adapter molecules CD14 (Wright et al., 1990) and MD-2 (Shimazu et al., 1999). TLR4 signaling might also therefore be fundamentally different in IECs, preventing what would be constant stimulation by the luminal Gram-negative bacterial population. Indeed, in cell culture of m-ICcl2 murine IECs, TLR4 was found to reside within the Golgi apparatus, rather than at the cell membrane (Hornef et al., 2002). In our studies, we found that LPS (1 mg/ml) instilled directly into the lumen of a ligated segment of small intestine for 1.5 hours did not cause apoptosis and cell shedding. Similarly, double-stranded RNA has failed to elicit apoptosis and shedding when administered orally (McAllister et al., 2013).

The rapid increase in plasma TNF concentration after LPS administration has been previously characterized (Copeland et al., 2005). In the current study, we also demonstrated a large fold change in *Tnf* mRNA abundance in small intestinal epithelial enriched extracts 1.5 hours after LPS administration. The significance of TNF as the crucial mediator in our model was further demonstrated by the fact that TNF administration caused very comparable IEC apoptosis and shedding as did LPS, consistent with previous results (Garside et al., 1993; Piguet et al., 1998). In the intestinal epithelium, and in intestinal cell lines, TNFR1 is expressed to a greater extent than TNFR2 (Lau et al., 2011; Mizoguchi et al., 2002), although the latter can be induced by inflammatory cytokines (Mizoguchi et al., 2002). TNFR1 has well-defined proapoptotic effects (Locksley et al., 2001) and, in our model, was essential for LPS-induced IEC apoptosis and shedding. This suggests that direct TNFR1 ligation occurs at the level of the IEC, although interactions in intermediary cell types, such as endothelial or myofibroblast cells, cannot be excluded. The role of TNFR2 has been less well characterized and interestingly the intestinal epithelial response to LPS was moderate in *Tnfr2^−/−^* mice, suggesting that this receptor does participate in LPS-induced IEC apoptosis, possibly by enhancing TNFR1 signals through degradation of TRAF2, resulting in termination of NFκB signaling (Rodríguez et al., 2011).

**Fig. 7.**
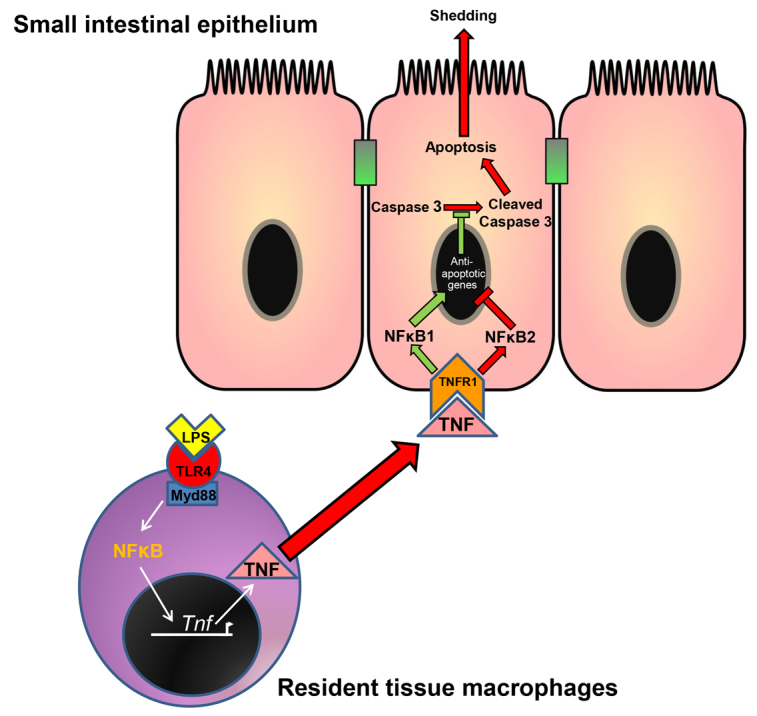
**Diagram summarizing the putative mechanism by which LPS induces apoptosis in IECs.** Systemically delivered LPS is first recognized predominantly by resident TLR4-expressing mononuclear cells (monocytes/macrophages/dendritic cells), which produce TNF. TNF is released into the systemic circulation and binds with TNFR1 on IECs, triggering apoptosis and shedding if NFκB2 signaling dominates, or cell survival if NFκB1 signaling dominates.

Mice deficient in NFκB1, an individual NFκB family member that signals via the canonical activation pathway, developed more IEC apoptosis and shedding in response to TNF than did WT mice. This is not surprising because TNFR1, as well as initiating apoptosis, also induces the expression of anti-apoptotic genes via NFκB (Wang et al., 1998). However, an interesting finding to emerge from our study was that mice deficient in NFκB2, an NFκB family member that signals via the alternative activation pathway, were more resistant to villus IEC apoptosis and shedding in response to TNF, compared with WT mice. This suggests that alternative NFκB2 pathway activation contributes to cell shedding and apoptosis. A possible mechanism to account for this effect could be due to the NFκB2 precursor acting as an inhibitor of NFκB activity (Basak et al., 2007; Fukushima et al., 2012). Absence of NFκB2 might therefore result in an extended expression of p65 (RelA)-induced anti-apoptotic genes (summarized in Fig. 7). The importance of p65 nuclear translocation in IECs that do not undergo shedding has been previously demonstrated in *Cryptosporidium parvum* infection (Foster et al., 2012). Alternatively, NFκB2-containing dimers might directly terminate the transcription of anti-apoptotic genes or induce the expression of proapoptotic genes.

In conclusion, we provide a detailed description of the early events and mechanisms that are responsible for acute, LPS-induced small intestinal injury. LPS therefore represents a robust, rapid and consistent stimulus for inducing pathological small intestinal epithelial cell apoptosis and shedding. Further study of this phenomenon could be highly relevant to our understanding of the initial pathogenesis of acute small intestinal disease states and diarrheal illnesses, and the intestinal manifestations of acute systemic disease states such as septic and endotoxic shock. Additionally, the very acute small intestinal lesions documented in this study might also contribute to development of chronic inflammation, such as is observed in IBD. Indeed, defective small intestinal permeability such as that which might occur in IEC shedding has been shown to contribute to the development of chronic colitis (Arrieta et al., 2009), meaning that this model might have relevance in CD or UC development. Ultimately, this could lead to the development of novel therapeutic strategies to ameliorate pathological villus epithelial cell apoptosis and shedding, and gut barrier dysfunction.

## MATERIALS AND METHODS

### Animals

Wild-type (WT) C57BL/6 mice supplied by Charles River (Margate, UK), and transgenic strains on a C57BL/6 background, including *Nfĸb1^−/−^* and *Nfĸb2^−/−^* mice (Caamaño et al., 1998; Sha et al., 1995) and *Tlr4^−/−^* mice (Hoshino et al., 1999) (generated by Shizuo Akira and supplied by Mark Taylor) were maintained at the University of Liverpool. *Tnfr1^−/−^* and *Tnfr2^−/−^* mice (Peschon et al., 1998) were maintained at the Saban Research Institute at Children’s Hospital Los Angeles. *Vil-Cre Myd88^−/−^* mice were maintained at the Disease Modelling Unit, University of East Anglia. All procedures were performed on adult mice (minimum age 9 weeks) under appropriate UK Home Office licenses or with approval and monitoring by the Children’s Hospital Los Angeles Institutional Animal Care and Use Committee.

### Generation of *Vil-Cre Myd88^−/−^* mice

*Myd88^fl/fl^*, which express a truncated mutant Myd88 protein following removal of the floxed region, were bred with *Vil-Cre* mice, which conditionally express Cre recombinase under control of the villin promoter. Offspring were genotyped for the presence of WT *Myd88*, mutated *Myd88* and *Cre* alleles. Mice were on a C57BL/6 genetic background.

### Lipopolysaccharide

LPS from *Escherichia coli* O111:B4 purified by phenol-extraction (PELPS) or ion-exchange chromatography (IE-LPS) (Sigma-Aldrich, Gillingham, UK) was diluted in sterile phosphate-buffered saline (PBS) and administered to mice by intraperitoneal (i.p.) injection.

### TNF

Murine recombinant TNF (Peprotech Ltd, London, UK) was diluted in sterile water and administered by i.p. injection to mice at 0.33 mg/kg body weight.

### Tissue processing

Following euthanasia, the intestinal tract was dissected *en bloc*. The intestinal lumina were flushed with PBS and immediately fixed in 10% neutral buffered formalin with selected organ samples. After 24 hours fixation, tissue was routinely processed and embedded in paraffin wax. Tissue sections (3–5 μm thickness) were prepared and stained either with H&E or used for immunohistochemistry (IHC).

### Immunohistochemistry for apoptotic IECs

Tissue sections were treated with 1% hydrogen peroxide in methanol to block endogenous peroxidases, followed by heat-induced antigen retrieval in 0.01 M citrate acid buffer (pH 6) and incubation with a rabbit polyclonal anti-active-caspase-3 antibody (AF835: R&D Systems, Abingdon, UK). Peroxidase-labeled anti-rabbit EnVision™ (Dako, Cambridge, UK) and 3,3′-diaminobenzidine were used for visualization.

### Quantification of active-caspase-3-positive cells

For quantification of apoptotic and shedding IECs, individual epithelial cells were counted from the base of the villus (above crypt level) to the mid-point of the villus tip in 18–20 well-orientated hemivilli at 400× magnification (delineated by red line in Fig. 2B). IECs were categorized according to the following criteria:

- normal’ if there was no or weak diffuse non-specific brown staining and cells had a basally located basophilic nucleus;
- apoptotic’ if there was defined positive staining that was confined to cytoplasmic or nuclear borders when compared with any background staining of neighboring IECs;
- shedding’ if there was defined positive staining that was confined to cytoplasmic or nuclear borders and in addition there was apical elevation of the cytoplasmic membrane, and/or an apically positioned nucleus.

Crypt IECs were counted from the crypt base to the crypt-villus junction in 19–20 well-orientated duodenal hemicrypts per mouse. Crypt IECs were simply categorized as ‘normal’ or ‘apoptotic’ because no discernible evidence of shedding was observed within crypts.

### Cell positional data

To allow comparison of cell positional data, villi were adjusted to a fixed length of 100 cells by using Wincrypts^©^ software (Cancer Research Campaign 1999). Data are then represented as percentage of villus length, 0% therefore representing the villus base and 100% representing the villus tip.

### Measurement of villus height

ImageJ (Schneider et al., 2012) was used to assess images captured by a Leica DMLA microscope, by setting the scale with a hemocytometer at 100× magnification. All images were captured at 100× magnification, and villi were measured by using the segmented line tool. Each segmented line was placed to originate at the base of the villus, above the level of adjoining crypts, and a segmented line extended to the villus tip, following any curvature of the villus. The mean of these segmented line lengths for ten well-orientated villi was calculated for each animal, and a mean value was then calculated for each group.

### Gut permeability assessment

Fluorescein-isothiocyanate-conjugated dextran (FD4: Sigma-Aldrich, Gillingham, UK) was diluted to 22 mg/ml in PBS and administered at 20 ml/kg body weight by oral gavage ± i.p. injection of LPS. Plasma fluorescence was measured by a TECAN Infinite^®^ F200 plate reader (excitation 485 nm, emission 535 nm) from blood collected post-mortem at 5 hours after gavage in order to allow FD4 to be distributed throughout the intestinal tract. Mice were euthanized via a rising CO_2_ concentration and blood taken by cardiac puncture. To assess the effect of LPS on permeability, it was administered at set time points prior to the end of the experiment, i.e. at 1.5 hours, 3 hours and at the same time as FD4 for the 5-hour time point. Plasma was separated from heparinized whole blood by centrifugation at 5000 rpm for 2 minutes in a minicentrifuge.

### Real-time PCR

Small intestinal extracts were isolated with chelation buffer solution as previously described (Flint et al., 1991), and RNA was isolated with a High Pure RNA Tissue Kit (Roche, Burgess Hill, UK). Reverse-transcription was performed with an RT^2^ reverse-transcription kit (SABiosciences, Crawley, UK). An 89 gene Cell-Death Pathway Finder array (SABiosciences) was performed on a Roche LightCycler^®^480 followed by validation with replicate samples using TaqMan^®^ gene expression assays for β-actin (Mm01205647_g1), TNF (Mm00443260_g1) and CD40 (Mm00441891_m1; Life Technologies, Paisley, UK). Cycling conditions were performed as per the manufacturer’s instructions.

### Data analysis

Data represent mean ± s.e.m. Comparisons were made between treatment groups and controls using SigmaPlot 12^©^ (Systat Software, London, UK). Normally distributed data were assessed by ANOVA with Holm-Sidak post-hoc test, and non-parametric data were analyzed by ANOVA on ranks (Kruskal-Wallis) with Dunn’s post-hoc test. *P*<0.05 was considered significant. REST^©^ software was used for comparison of qPCR data from individualized samples by randomization test as previously described (Pfaffl et al., 2002). *n* numbers indicate the total number of mice studied.

## Supplementary Material

Supplementary Material
