# Supplementary material for: A mouse model of pathological small intestinal epithelial cell apoptosis and shedding induced by systemic administration of lipopolysaccharide
Source: Dis Model Mech. 2013 Aug 15;6(6):1388–99. doi: 10.1242/dmm.013284 (PMC3820262; doi:10.1242/dmm.013284)
Supplement: Supplementary Material [file supp_6_6_1388__index.html]

A mouse model of pathological small intestinal epithelial cell apoptosis and shedding induced by systemic administration of lipopolysaccharide — Supplementary Material 

# A mouse model of pathological small intestinal epithelial cell apoptosis and shedding induced by systemic administration of lipopolysaccharide

## DMM013284 Supplementary Material

**Files in this Data Supplement:**

- **Supplementary Material PDF**
