# Supplementary material for: A mouse model of pathological small intestinal epithelial cell apoptosis and shedding induced by systemic administration of lipopolysaccharide
Source: Dis Model Mech. 2013 Aug 15;6(6):1388–99. doi: 10.1242/dmm.013284 (PMC3820262; doi:10.1242/dmm.013284)
Supplement: Supplementary Material [file supp_013284_DMM013284.pdf]

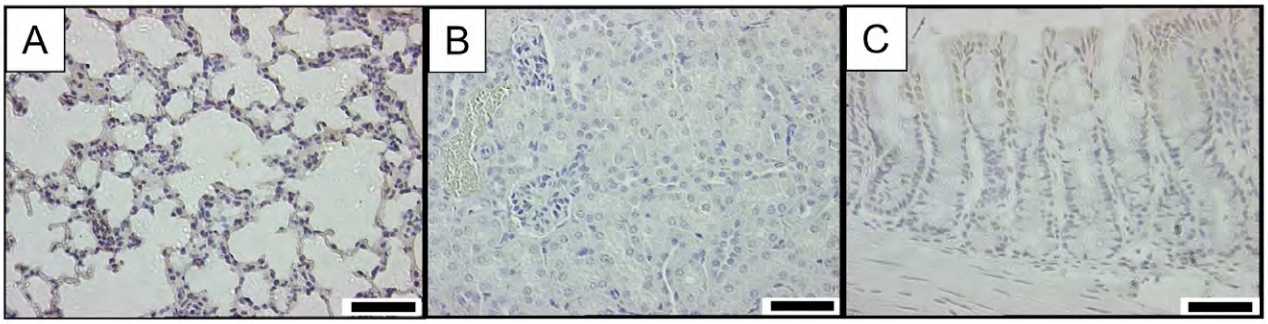

Supplementary Figure 1: Other target organs commonly damaged in endotoxic/septic shock did not show evidence of cell death. IHC for active Caspase-3 shows lack of immunolabelling in lung (A), kidney (B), or other localizations of the gastrointestinal tract; colon(C) of 1.5h 10mg/kg LPS treated mice with confirmed apoptosis in the small intestine (bars=50µm).

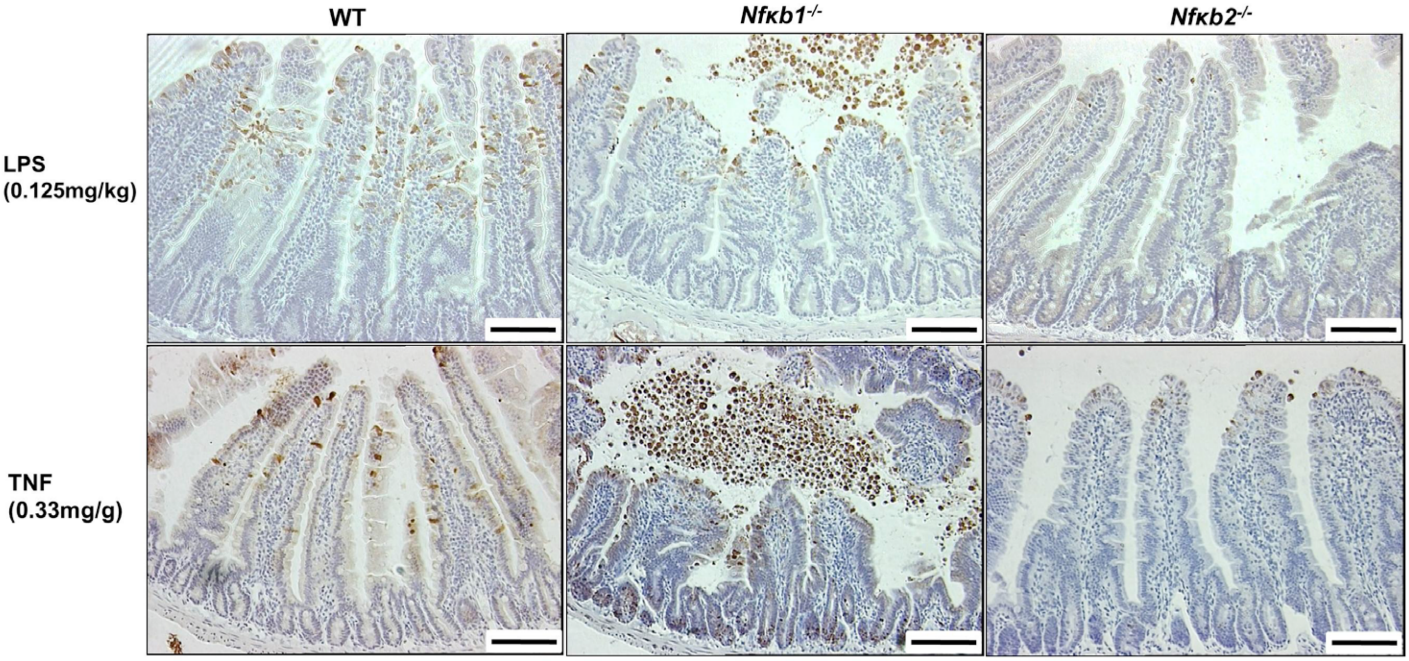

Supplementary Figure 2: ***Nfκb1***$^{-/-}$ **are more sensitive and** ***Nfκb2***$^{-/-}$ **more resistant to LPS or TNF induced IEC apoptosis and shedding.** Representative photomicrographs of LPS or TNF treated female WT, *Nfκb1*$^{-/-}$, and *Nfκb2*$^{-/-}$ showing the enhanced apoptosis/shedding and villus shortening in *Nfκb1*$^{-/-}$ and reduced apoptosis/shedding and villus shortening in *Nfκb2*$^{-/-}$. Bars=100µm.

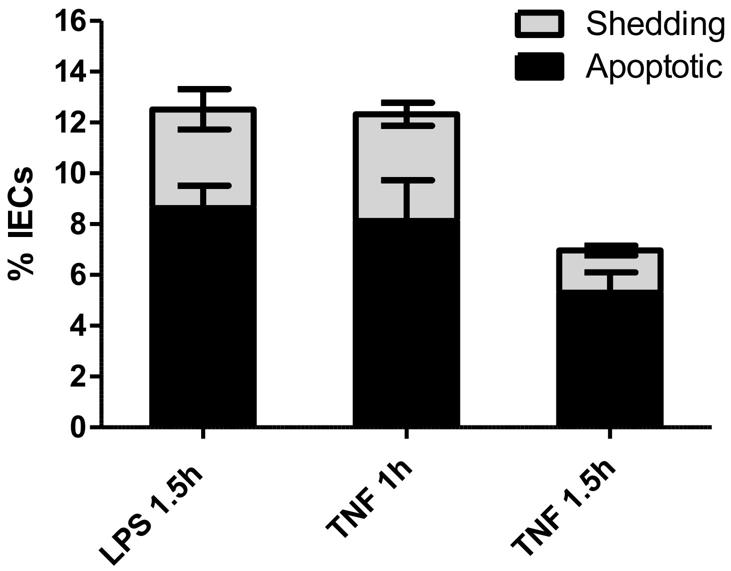

Supplementary Figure 3: TNF caused more rapid small intestinal apoptosis and shedding than LPS. TNF (0.33mg/g) caused very similar amounts of apoptosis and IEC shedding at 1h to that caused by 10mg/kg PE-LPS at 1.5h. By 1.5h, maximum apoptosis and shedding had subsided in TNF treated animals (n=4-6 C57BL/6 female mice/group).
